# Some Ancient Anecdotes

**Published:** 1886-09

**Authors:** 


### *SOME ANCIENT ANECDOTES.*

*From the Journal D'Hygiene.*

**A sick person** upon being asked why he did not call in a physician, replied, "Because I am not yet anxious to die."

**A physician** upon hearing those of his profession spoken ill of, said, "No one complains after being attended by me." "No," was the reply, "for you kill all you treat."

**The Romans,** having banished from Rome Greek physicians, who distinguished themselves, for the most part, by the number of deaths, Canton was led to make this reflection: "The Greeks, jealous of the glory of the Romans, being unable to vanquish them upon the field of battle, have sent their executioners to assassinate them in their beds."

**If you require** any medicines, says the College of Salerne, let them be these three, which are always at hand: "A bright and peaceful mind, a plain diet, and a moderate exercise." Dumoulin also held the same opinion. This celebrated physician in his last hours, being surrounded by many physicians of Paris, who deplored his approaching end, said to them, "Gentlemen, I leave behind me three great physicians." Each one in attendance, believing himself to be one of the three, urged Dumoulin to name them, whereupon he replied, "*Water*, *exercise* and *diet*."

**Moliere's** theatrical plays make physicians a subject of ridicule. Among other sarcasms which he launches against them is the story of a Huguenot minister whose functions were prohibited by a conclave of his enemies; upon which he said haughtily, that this should cost the lives of more than a hundred men. Upon being cited before the judge for these words he explained himself by saying that if they interdicted his ministry he would turn physician. This fact is introduced in the comedy "du Grondeur." This personage, who is a physician, provoked at the consummation of the marriage of Mondor with his daughter contrary to his will, wrote in his anger, "This will cost many lives."

